# 101. Serious Adverse Event Rates for Cefazolin and Ceftriaxone Outpatient Parenteral Antimicrobial Therapy

**DOI:** 10.1093/ofid/ofad500.017

**Published:** 2023-11-27

**Authors:** Shawnalyn Sunagawa, Sarah Arduser, Molly M Miller, Elizabeth Lyden, Nicolas W Cortes-Penfield, Richard Hankins, Scott J Bergman, Bryan T Alexander

**Affiliations:** Nebraska Medicine, Omaha, Nebraska; Nebraska Medicine, Omaha, Nebraska; Nebraska Medicine, Omaha, Nebraska; University of Nebraska Medical Center, Omaha, Nebraska; University of Nebraska Medical Center, Omaha, Nebraska; Nebraska Medicine, Omaha, Nebraska; Nebraska Medicine, Omaha, Nebraska; Nebraska Medicine, Omaha, Nebraska

## Abstract

**Background:**

Beta-lactams are thought to be well-tolerated in outpatient parenteral antimicrobial therapy (OPAT), but few studies address the utility of regularly monitoring laboratory parameters for beta-lactams in the OPAT setting. Our aim was to assess OPAT adverse event (AE) rates for cefazolin and ceftriaxone to better define appropriate laboratory monitoring for these antimicrobials.

**Methods:**

We retrospectively evaluated patients prescribed either cefazolin or ceftriaxone via OPAT between 3/1/2019-9/30/2022. Combination intravenous antimicrobial therapies were excluded. The primary outcome was incidence of clinically significant OPAT-related AEs, defined as drug-related AE leading to treatment alterations (e.g., abnormal labs, allergic reactions, *Clostridioides difficile* infection) or any catheter-associated AE. Secondary outcomes included time from start of therapy to clinically significant AEs, unplanned healthcare utilization (e.g., emergency department visits, readmissions), and factors associated with increased risk of clinically significant AEs. We performed descriptive statistics for cohort characteristics, Fisher’s exact and independent sample T-tests for associations with primary and secondary outcomes, and univariate and multivariate regressions for predictors of AEs.

**Results:**

Characteristics of the cohort are listed in Table 1. Primary and secondary outcomes are reported in Table 2. There were 3282 sets of weekly labs, 99.7% were obtained and reviewed within 72 hours from lab draw. Laboratory monitoring detected 2.7 clinically significant AEs per 1000 sets of weekly labs. Median time from start of therapy to abnormal lab drug-associated AE was 26 days (IQR 19, 34). Univariate and multivariate logistic regressions are listed in Table 3. There were no statistically significant differences in antibiotic, duration of therapy, or OPAT modality between the groups with or without clinically significant drug-related AE.

**Figure:**
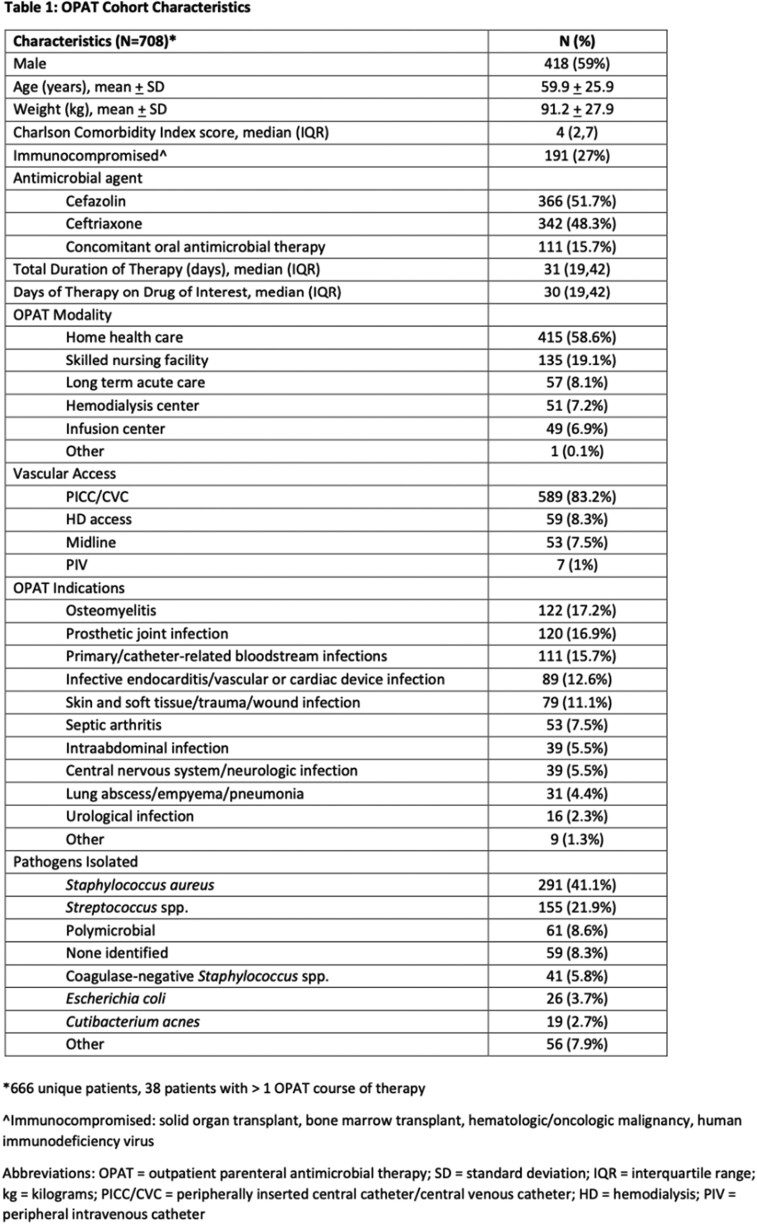

**Figure:**
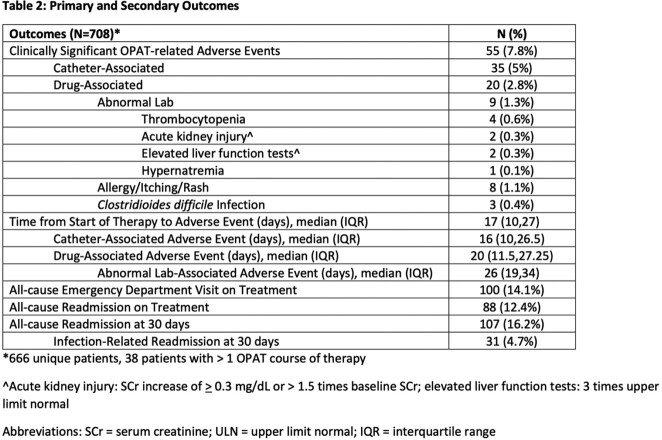

**Figure:**
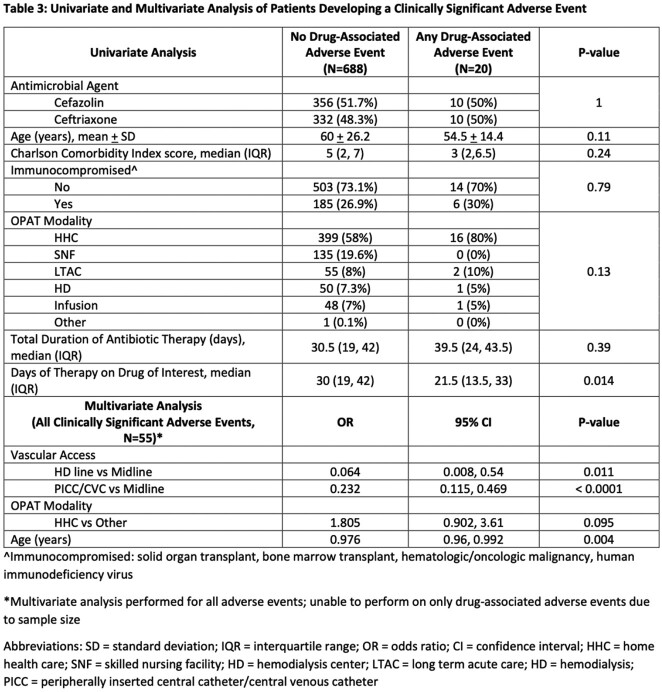

**Conclusion:**

Cefazolin and ceftriaxone were well tolerated during OPAT, with few drug-associated AEs mainly occurring beyond 3 weeks of therapy. As we identified just 2.7 clinically significant AEs per 1000 sets of weekly labs, routine weekly laboratory monitoring for cefazolin and ceftriaxone in the OPAT setting may be excessive.

**Disclosures:**

**Scott J. Bergman, PharmD**, bioMerieux, Inc.: Honoraria **Bryan T. Alexander, PharmD, BCIDP, AAHIVP**, Astellas Pharma: Advisor/Consultant|F2G: Advisor/Consultant|Merck: Grant/Research Support

